# Gamma Radiation-Induced Synthesis of Carboxymethyl Cellulose-Acrylic Acid Hydrogels for Methylene Blue Dye Removal

**DOI:** 10.3390/gels10120785

**Published:** 2024-12-01

**Authors:** Sabuj Chandra Sutradhar, Nipa Banik, Mobinul Islam, Mohammad Mizanur Rahman Khan, Jae-Ho Jeong

**Affiliations:** 1 Department of Energy & Materials Engineering, Konkuk University, Chungju-si 27478, Republic of Korea; chandra@kku.ac.kr; 2 Department of Chemical and Biological Engineering, College of Engineering, Korea National University of Transportation, Chungju-si 27469, Republic of Korea; baniknipa@gmail.com; 3 Department of Energy & Materials Engineering, Dongguk University, Seoul 04620, Republic of Korea; mobin85@dongguk.edu; 4 Research Center for Green Energy Systems, Department of Mechanical Engineering, Gachon University, Seongnam-si 13120, Republic of Korea

**Keywords:** methylene blue (MB), CMC-based hydrogels, gamma radiation, pseudo-second-order model, intraparticle diffusion, film diffusion

## Abstract

This study aims to develop efficient and sustainable hydrogels for dye adsorption, addressing the critical need for improved wastewater treatment methods. Carboxymethyl cellulose (CMC)-based hydrogels grafted with AAc were synthesized using gamma radiation polymerization. Various AAc to CMC ratios (5:5, 5:7.5, 5:10, 5:15) were treated with 37% NaOH and exposed to 1–15 kGy radiation, with the optimal hydrogel obtained at 5 kGy. Swelling studies showed an increase in swelling with 5–7.5% AAc content, with the 5:7.5 hydrogel achieving the highest swelling at 18,774.60 (g/g). FTIR spectroscopy confirmed the interaction between AAc and CMC, indicating the successful formation of the hydrogel. DSC analysis revealed that higher AAc content led to increased glass transition and decomposition temperatures, thereby enhancing thermal stability. The swelling kinetics were linked to a reduction in pore size and improved AAc grafting. The 5:7.5 hydrogel demonstrated the highest adsorption capacity (681 mg/g) for methylene blue at 80 mg/L, achieving a desorption efficiency of 95% in 2M HCl. Kinetic analysis revealed non-uniform physisorption on a heterogeneous surface, which followed Schott’s pseudo-second-order model. This study advances the development of efficient hydrogels for water purification, providing a cost-effective and environmentally friendly solution for large-scale applications.

## 1. Introduction

The persistent issue of water pollution, particularly from synthetic dyes like methylene blue (MB), poses a significant threat to environmental and human health [1,2]. Developing efficient and sustainable wastewater treatment methods is critical due to increasing industrial effluents. MB and similar dyes are particularly concerning due to their toxicity and persistence [3]. Consequently, removing these dyes from aqueous solutions has become a major research focus [4,5].

Currently, dye wastewater is often treated using physical, chemical, and biological treatment techniques [6,7,8]. However, these techniques frequently involve high energy consumption, high expenses, and the generation of hazardous byproducts. Among these approaches, adsorption stands out as a promising approach due to its affordability, environmental friendliness, simplicity, and high effectiveness [9,10].

Materials commonly used in adsorption include activated carbon [11], metal oxides [12] biomass-based materials [13] synthetic [14] and modified natural polymers [15], and zeolites [16]. Although adsorption shows great potential, it faces several challenges, such as low capacity of native materials, complex dye and heavy metal structures, and varying environmental conditions such as pH, temperature, and pollutant concentration [17]. Additionally, factors like cost, availability, regeneration, reuse, and disposal of adsorbents significantly impact the efficiency and feasibility of adsorption for wastewater treatment [18].

Hydrogels, characterized by their cross-linked polymer structure and high-water content, are particularly effective for dye adsorption. Their ability to be regenerated and reused makes them a sustainable choice for wastewater treatment [19,20].

CMC, a cellulose derivative, is especially suitable for hydrogel synthesis due to its water solubility, biocompatibility, and high-water retention capacity. Chemical modifications, such as adding hydroxyl and carboxyl groups, enhance their adsorption properties [21].

Recent studies have extensively explored the use of CMC as an efficient adsorbent for the removal of MB from wastewater. For example, Sabarish Radoor et al. [22] demonstrated that a PVA/CMC/halloysite nano clay biocomposite had an adsorption capacity of 125.8 mg/g and a removal efficiency of 98.6%. Mostafa Ahmed Ibrahim et al. [23] reported a cellulose nanocrystals/CMC/zeolite membrane with an adsorption capacity of 76.5 mg/g and a removal efficiency of 95%, emphasizing adsorption kinetics and thermodynamics. Similarly, Chen et al. [24] developed a sugarcane cellulose-based composite hydrogel enhanced with g-C_3_N_4_ nanosheets for selective organic dye removal, achieving a high adsorption capacity of 362.3 mg/g for MB while maintaining efficiency over multiple cycles. Ma Q. et al. [25] developed a hydrogel based on carboxymethyl cellulose, incorporating two-dimensional montmorillonite nanosheets (CMC-MMTs Gel) for MB removal, showing an impressive adsorption capacity of 410 mg/g, increased porosity, and sustained performance over five cycles, indicating excellent reusability. Additionally, Liang Shi et al. [26] demonstrated a cross-linked carboxymethyl cellulose/organo-montmorillonite composite hydrogel with a remarkable adsorption capacity of 490.5 mg/g.

Recent studies have highlighted the versatility and effectiveness of CMC-based materials in dye removal applications, enhancing our understanding of the parameters governing adsorption performance [27]. These findings underscore the potential of CMC as a sustainable solution for wastewater treatment. However, gaps remain in optimizing the selectivity and adsorption capacity of CMC-based adsorbents, particularly in complex industrial wastewater containing multiple contaminants [28]. Furthermore, challenges such as the poor mechanical strength, water solubility of CMC long-term stability, and reusability of these adsorbents require further investigation [29].

However, functionalizing CMC with synthetic polymers can enhance these properties, thereby improving both mechanical strength and adsorption capacity [30]. Gamma radiation can synthesize hydrogels by inducing cross-linking without chemical initiators, enhancing structural properties. This technique facilitates the formation of highly cross-linked, stable hydrogels with enhanced mechanical strength and swelling capacity, thereby enhancing adsorption efficiency for pollutants like MB dye [31,32]. Additionally, the incorporation of acrylic acid (AAc) into hydrogels further enhances adsorption properties by providing additional functional groups, improving efficiency and selectivity for MB dyes [33].

Despite the promising potential of gamma-radiated hydrogels for dye adsorption, several research gaps persist, including optimizing radiation dosage and understanding competitive adsorption in the presence of multiple contaminants.

This study addresses gaps by synthesizing gamma-radiated CMC/AAc blend hydrogels with enhanced MB dye adsorption efficiency and capacity. It explores synthesis, optimizes radiation dosage, characterizes properties, evaluates adsorption efficiency, environmental friendliness, and cost-effectiveness, investigates competitive adsorption behavior, and assesses the hydrogels’ effectiveness in removing MB dye from aqueous solutions, providing valuable insights for wastewater treatment applications.

## 2. Results and Discussion

### 2.1. Reaction Pathway in Gamma-Irradiated CMC/AAc Hydrogels

**Scheme 1:**
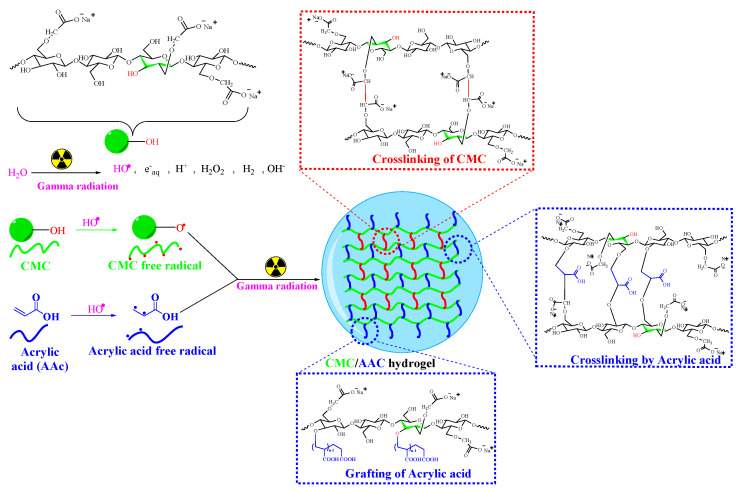
Possible grafting and cross-linking reaction mechanism of CMC/AAc hydrogel.

The process of polymer cross-linking in solution via γ-irradiation has been widely explored [34,35]. This study involved synthesizing a hydrogel from carboxymethyl cellulose by grafting it with acrylic acid using gamma irradiation in an aqueous medium. The proposed mechanism of network formation is illustrated in Scheme 1.

CMC is a linear polymer composed of anhydroglucose units linked by β-glycosidic bonds at C-1 and C-4, featuring numerous hydroxyl groups. When the aqueous blend of CMC and AAc is exposed to gamma irradiation, it generates three main reactive species: hydrated electrons, hydroxyl radicals, and hydrogen radicals [36]. Hydrated electrons are minimally reactive with hydrophilic hydrogel-forming polymers. In acidic media, hydrogen radicals contribute about 20% to macroradical yield. Hydroxyl radicals, generated from water, extract hydrogen atoms from CMC macromolecules, resulting in the formation of macroradicals (as shown in Scheme 1) [37]. Gamma radiation also initiates the free radical polymerization of AAc on the CMC backbone [38]. Studies indicate that in cellulose derivatives like CMC, free radicals are generated at substituted side chains, which are responsible for grafting or intermolecular cross-linking [39]. A stable radical associated with the α-carbon atom of the substituent group, RO.CH–COO–, plays a role in intermolecular cross-linking. Therefore, irradiation of both CMC and AAc produces free radicals that combine to form the hydrogel.

### 2.2. FTIR Spectra Analysis

**Figure 1:**
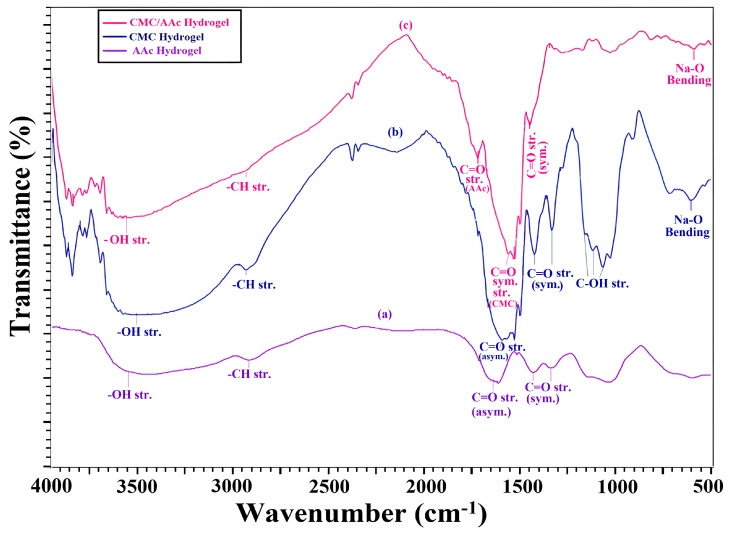
FTIR spectra of (a) acrylic acid (AAc), (b) carboxymethyl cellulose (CMC), (c) CMC/AAc hydrogel.

FTIR analysis was conducted to reveal bond formation and cross-linking within anhydroglucose units. Figure 1 displays the FTIR spectra of AAc, pure CMC powder, and the CMC/AAc hydrogel (spectra a, b, and c).

In the FTIR spectrum of polyacrylic acid (PAAc) (spectrum 1a), the stretching frequencies at 3550.25–2403.02 cm^−1^ and 1650 cm^−1^ correspond to the O-H and carbonyl groups of carboxylic acid, respectively.

The FTIR spectrum of CMC (Figure 1b) exhibited a broad peak at 3126–3606 cm^−1^ for O-H stretching vibrations. The C-H stretching can be seen around 2926.01 cm^−1^, while the band at 1060.85 cm^−1^ corresponds to C-O-C stretching. The peak at 1585.49 cm^−1^ is attributed to asymmetric COO^−^ stretching, with smaller peaks at 1417.68 cm^−1^ and 1325 cm^−1^ for symmetric COO stretching [40].

Upon grafting AAc to the CMC backbone (Figure 1c), the characteristic absorption bands of CMC at 1060.85, 1112.93, and 1151.50 cm^−1^, which are associated with C-OH stretching vibrations, were significantly diminished due to the formation of CHO-CH_2_ groups from the grafting reaction between the hydroxyl group at the C2 position of anhydroglucose and the CH_2_- of AAc.

In the IR spectrum of CMC/AAc graft polymer hydrogels, additional peaks were observed around 1714.72 cm^−1^ indicating C=O. The peak at approximately 3572.17 cm^−1^ in the CMC/AAc polymer hydrogels represents the overlap of hydroxyl groups from both CMC and AAc. Additionally, the intense asymmetric stretching vibration of COO^−^ groups in CMC at 1585.49 cm^−1^ shifted to 1523.76 cm^−1^, overlapping with the characteristic absorption of COO^−^ groups of grafted AAc chains, forming a broad band [41]. The metal-oxygen (Na-O) bending vibration in CMC/AAc hydrogels appeared around 590 cm^−1^, originating from the CMC powder before gamma radiation with AAc. Furthermore, the sharp peak around 1442.75 cm^−1^, corresponding to the symmetric stretching mode of the carboxylate anion, indicates successful grafting of AAc onto the CMC backbone.

### 2.3. Thermal Analysis of Gamma-Radiated CMC/AAc Hydrogels

**Figure 2:**
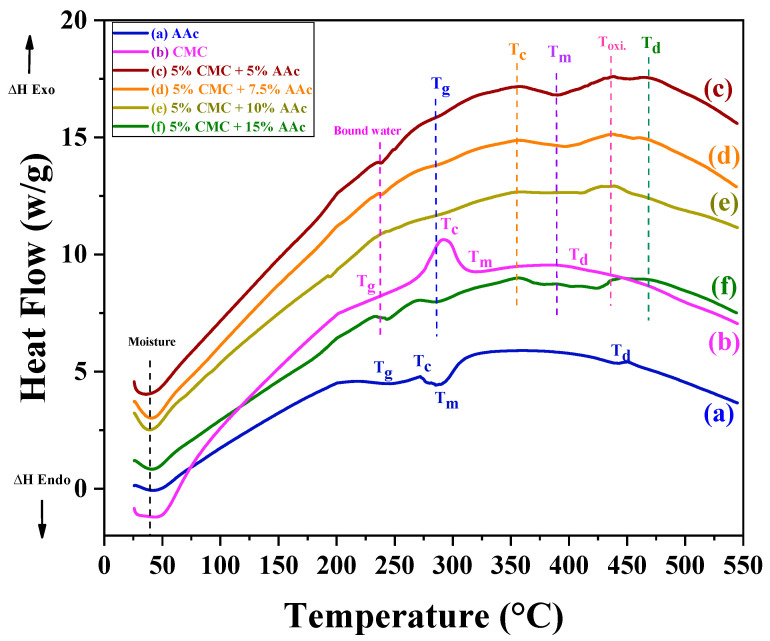
Differential scanning calorimetry (DSC) analysis of CMC/AAc hydrogels.

The thermal behaviors of gamma-radiated CMC/AAc polymer hydrogels were analyzed using differential scanning calorimetry (DSC), revealing significant changes with increasing AAc content and cross-linking density. The DSC curves for CMC and cross-linked CMC are presented in Figure 2, with thermal transition temperatures listed in Table 1.

For CMC/AAc cross-linked hydrogels, two stages of water removal were observed: the removal of free water below 50 °C and the vaporization of bound water below 250 °C. This behavior can be attributed to the formation of hydrogen bonds between sodium carboxymethyl groups (Na^+^/CMC^−^) and water molecules [42].

Pure CMC and AAc samples exhibited endothermic glass transition peaks (Tg) at 228.73 °C and 242.01 °C, respectively, alongside exothermic peaks at 293.73 °C and 272.05 °C due to crystallization (Tc).

The Tg of CMC/AAc hydrogels shifts to higher temperatures (approximately 285.34 °C to 292.82 °C) as AAc content increases. Notably, Tg rises to 292.82 °C for a composition with 15% AAc cross-linked with 5% CMC. This shift is attributed to the restricted thermal motion of the polymer and the cross-linking of AAc with the rigid anhydroglucose unit of CMC [43]. The relatively weak and broadened glass transitions can be attributed to the semicrystalline nature of the material.

Similarly, the crystallization temperature (Tc) and melting point (Tm) increase with higher AAc content and cross-linking density, which is a result of enhanced hydrogen bonding and improved thermal stability [44]. The degradation temperature (Td) indicates that AAc not only raises thermal transition temperatures but also decreases the degradation rate, reflecting the enhanced thermal resistance of AAc cross-linked CMC hydrogels [45].

These findings highlight how the content of AAc and the degree of cross-linking affect the thermal behavior of gamma-radiated CMC/AAc hydrogels, providing valuable insights for their potential applications.

### 2.4. Biodegradability of CMC/AAc Hydrogel Films

The biodegradability of CMC/AAc hydrogel films was evaluated using a soil-burial test in a controlled composting environment. Conditions were maintained at 27–30 °C, with a pH of 6.5–8.0, and approximately 60% moisture content to support microbial growth. The hydrogel films measuring 55 mm in diameter, and 0.32–0.35 mm thickness, were buried for six weeks, with weekly assessments of morphological changes and weight loss.

**Figure 3:**
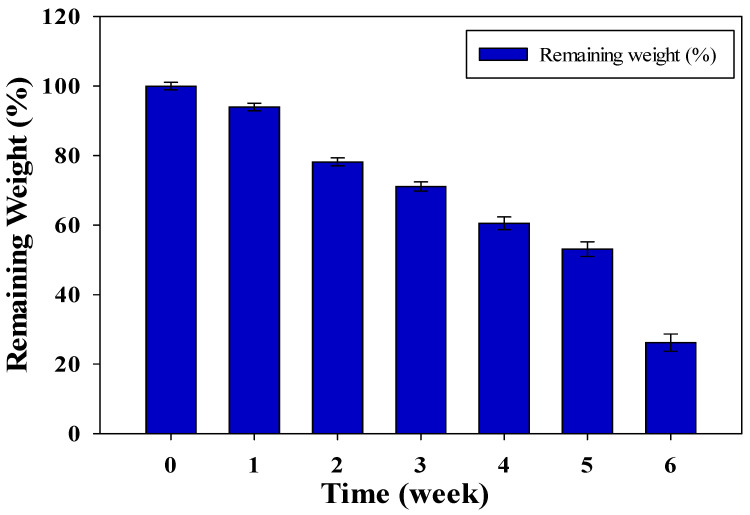
Weight loss profile of CMC/AAc hydrogel film under compost environment.

During the composting period, the weight of the CMC/AAc hydrogel films decreased significantly (as shown in Figure 3). By the end of the six weeks, only 26% of the hydrogel film recoverable residues remained, indicating rapid degradation.

### 2.5. Impact of Radiation and AAc Content on Grafting in CMC/AAc Hydrogels

**Figure 4:**
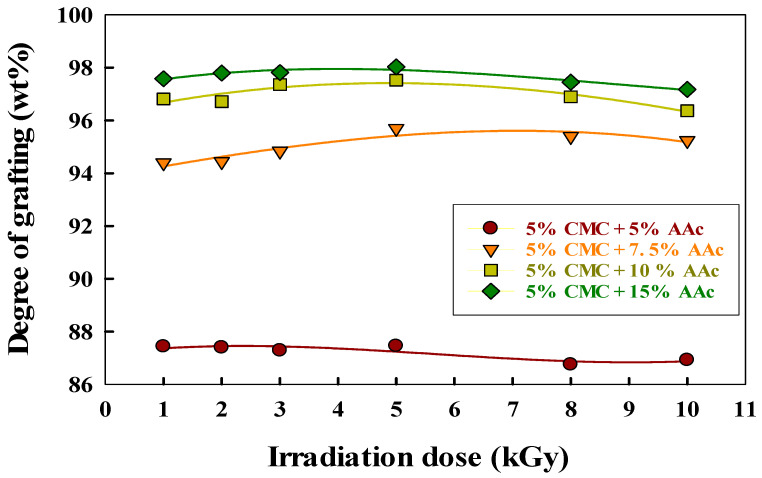
Effect of irradiation dose and CMC/AAc composition on grafting.

Figure 4 illustrates how the radiation dose and AAc concentration affect the grafting degree of CMC/AAc hydrogels.

The degree of grafting increased with radiation dose, peaking at 5 kGy, after which gel content stabilized. This plateau is due to cross-linking saturation, chain scission, steric hindrance, and radical recombination, which together limit further grafting despite increased radiation doses [46]. Additionally, Figure 4 also shows that the degree of grafting rose from 86.75% to 98.03% as AAc content increased from 5% to 15%. This increase occurred because a higher AAc concentration in the CMC/AAc blend hydrogel generated more free radicals during gamma irradiation, which are essential for initiating cross-linking with the CMC backbone. Consequently, more AAc leads to the production of more radicals, thereby enhancing gel content [47].

### 2.6. Impact of Acrylic Acid Concentration on CMC/AAc Hydrogel Properties

**Figure 5:**
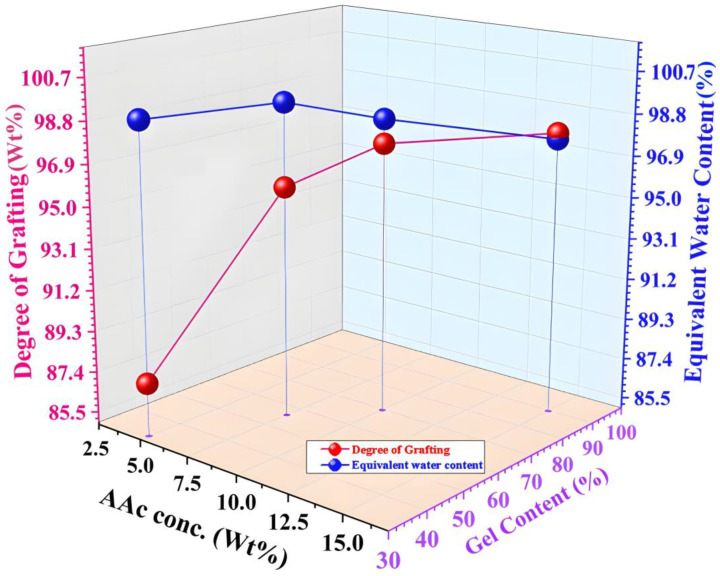
Effect of acrylic acid concentration on the degree of grafting (Wt%), gel content (%), and EWC (%) of CMC/AAc blend hydrogels at 5 kGy radiation dose.

Figure 5 demonstrates the impact of varying acrylic acid (AAc) levels on the grafting degree, gel fraction, and water content in CMC/AAc hydrogels exposed to a 5 kGy radiation dose.

The gel fraction and degree of grafting both rose from 32.13% to 89.59% and 87.46% to 98.03%, respectively, as AAc increased from 5.0% to 15.0%. During gamma irradiation, AAc generates free radicals, which enhance both grafting and gel content. However, water content increases from 5.0% to 7.5% AAc but decreases when the AAc concentration rises from 10.0% to 15.0% [48].

This observed behavior can be attributed to the density of cross-linking. The CMC and AAc chains form a network through grafting copolymers. The hydrophilic groups within these networks allow for the absorption and retention of water. According to Flory’s theory, there is an optimal cross-linking density that maximizes water absorbency [49]. Below this optimal level, water absorbency increases with cross-linking. Beyond it, water absorbency decreases as cross-linking density increases [50].

### 2.7. Effect of Standing Time on Water Absorption

**Figure 6:**
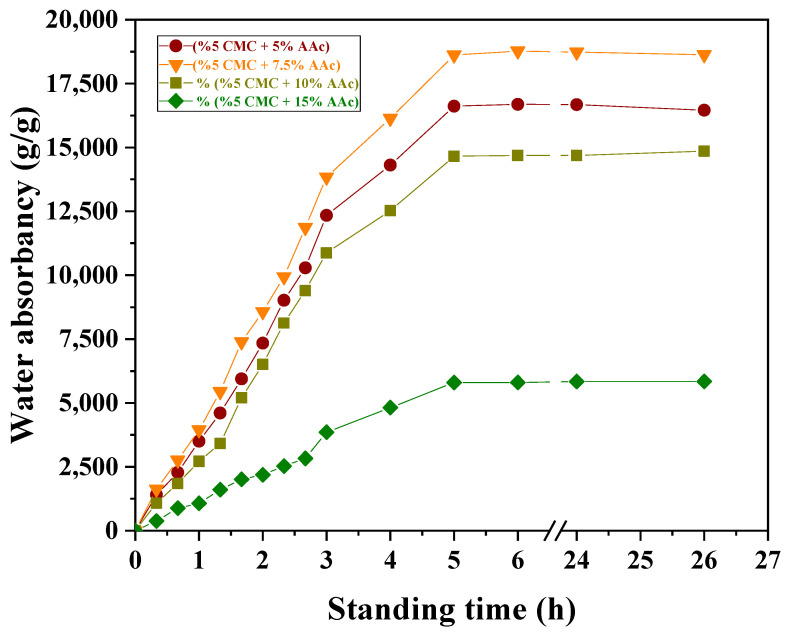
Effect of standing time and AAc concentration on water adsorption of CMC/AAc hydrogels at 5 kGy radiation dose.

Figure 6 illustrates the swelling rates of various partially neutralized CMC/AAc hydrogel compositions in distilled water at a 5 kGy radiation dose. The figure emphasizes how copolymeric composition, cross-linking density, and hydrogen bonding influence equilibrium swelling and water absorbency.

The equilibrium swelling for neutralized CMC/AAc (5/7.5) reached 18774.60 (g/g) at 24 h, demonstrating that the hydrophilic nature of the hydrogel significantly affects its swelling properties. Increased hydrophilicity accelerates swelling in an aqueous medium.

The observed decrease in swelling with increasing AAc content, with maximum swelling at (5/7.5) CMC/AAc, can be explained by the relationship between cross-linking density and water absorbency. When the cross-linking density was below the optimal level, water absorbency increased with the degree of cross-linking. However, beyond the optimal cross-linking density, water absorbency decreased as the network became too dense, restricting water uptake.

The swelling of CMC/AAc hydrogels was further reduced by hydrogen bonding between the hydroxyl groups of CMC and the free carboxylic groups of AAc. This bonding probably creates a robust network that regulates chain expansion and water diffusion [51]. Additionally, strong inter-chain hydrogen bonds between the CMC backbone and PAAc side chains contribute to reduced swelling [52].

### 2.8. Evaluation of CMC/AAc Hydrogels for Dye Removal from Wastewater

**Figure 7:**
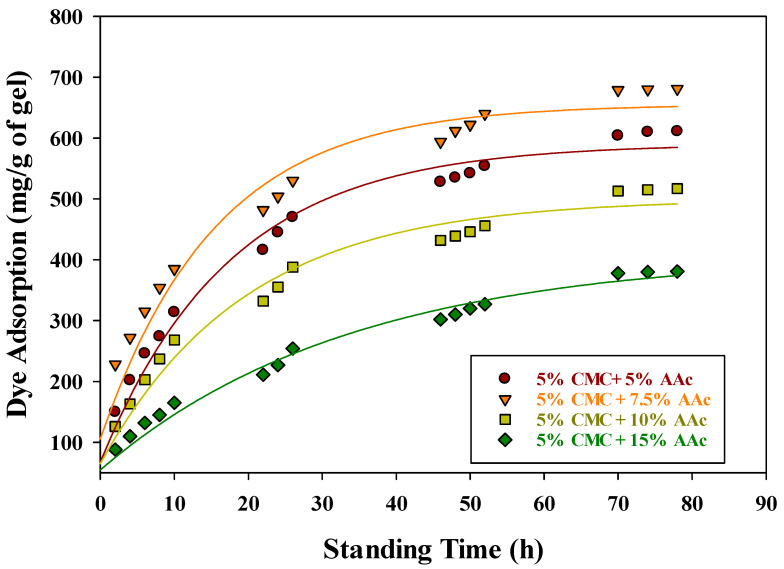
Effect of standing time and concentration of CMC/AAc hydrogel on dye adsorption.

The primary objective of this study, beyond examining the basic properties, was to evaluate the efficacy of CMC/AAc hydrogels in removing hazardous dyes from wastewater, using methylene blue (MB) as a model compound. For the MB adsorption experiments, CMC/AAc hydrogels synthesized with a 5 kGy gamma radiation dose were selected due to their optimal gel fraction. Figure 7 depicts the MB adsorption kinetics of CMC/AAc hydrogels with varying compositions, all prepared using a 5 kGy gamma radiation dose.

The adsorption capacity increased over time, reaching maximum values of approximately 611, 681, 517, and 381 mg/g for hydrogels with compositions of 5% CMC/5% AAc, 7.5% AAc, 10% AAc, and 15% AAc, respectively, after 78 h. Notably, the 5% CMC/7.5% AAc hydrogel demonstrated the highest MB adsorption efficiency, likely due to more favorable conditions for complex formation. The adsorption mechanism involves the COO^−^ groups of the CMC/AAc hydrogels forming an ionic complex with the imine groups of MB. Figure 7 also indicates that the initial MB adsorption rates for all CMC/AAc hydrogels were higher compared to the later stages. This trend can be attributed to two factors: the reduction in available –COO^−^ groups in the hydrogel [53] and the stiffening of the hydrogel network, which hinders the mobility of –COO^−^ groups in seeking out imine groups of MB for complex formation [54].

### 2.9. Adsorption Kinetics of MB on CMC/AAc Hydrogels

The transport rate of MB from the bulk solution to the surface of the adsorbent plays a crucial role in determining the adsorption kinetics. This process provides insights into the adsorption mechanism and the pathways of the reaction. Kinetic experiments were conducted using a 250 mL MB solution (80 mg/L) at 298 K. Samples were collected every 2 h for a duration of up to 78 h. The MB sorption mechanism on CMC/AAc hydrogels was investigated by fitting the experimental kinetic data to pseudo-first-order [55] pseudo-second-order [56] and Elovich kinetic model [57].

#### 2.9.1. Pseudo-First Order Kinetics

The linearized form of the pseudo-first-order model is represented by the following equation:

(1) $$\log\left(Q_{e}-\;Q_{t}\right)={\log Q}_{e}-\frac{k_{1}}{2.303}t$$

**Figure 8:**
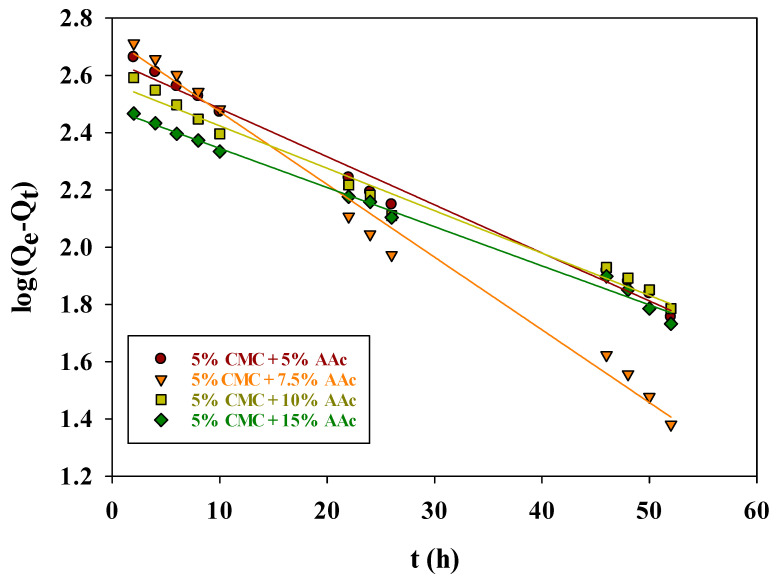
Pseudo-first-order kinetic model for adsorption of MB on CMC/AAc hydrogel.

The pseudo-first-order rate constant k_1_ (h^−1^) and the amount of adsorbate Q_t_ (mg/g) on the adsorbent surface at a given time (t) were derived from the linear plots of log (Q_e_ − Q_t_) vs. t (Figure 8).

These results, including the calculated equilibrium concentration Q_e_, _cal,_ and correlation coefficients (R^2^), are summarized in Table 2. The R^2^ values for the first-order kinetic model were 0.9865, 0.9895, 0.9831, and 0.9934 for 5%, 7.5%, and 15% AAc concentrations, respectively. The calculated (Q_e_) values (448.85, 533.84, 373.34, and 303.46 mg/g) differed from the experimental Q_e_ values (611, 681, 517, and 381 mg/g), indicating the pseudo-first-order model was inadequate for describing MB sorption on CMC/AAc hydrogels.

#### 2.9.2. Pseudo-Second Order Kinetics

The pseudo-second-order rate model depends on the number of adsorption sites and the concentration of adsorbate ions in the liquid phase. The linearized form of this kinetic model is expressed as follows:

(2) $$\frac{t}{Q_{t}}=\frac{1}{k_{2}{Q_{e}}^{2}}+\frac{t}{Q_{e}}$$

In this equation, (Q_e_), (Q_t_), and (t) retain their usual definitions, while (k_2_) is the rate constant.

**Figure 9:**
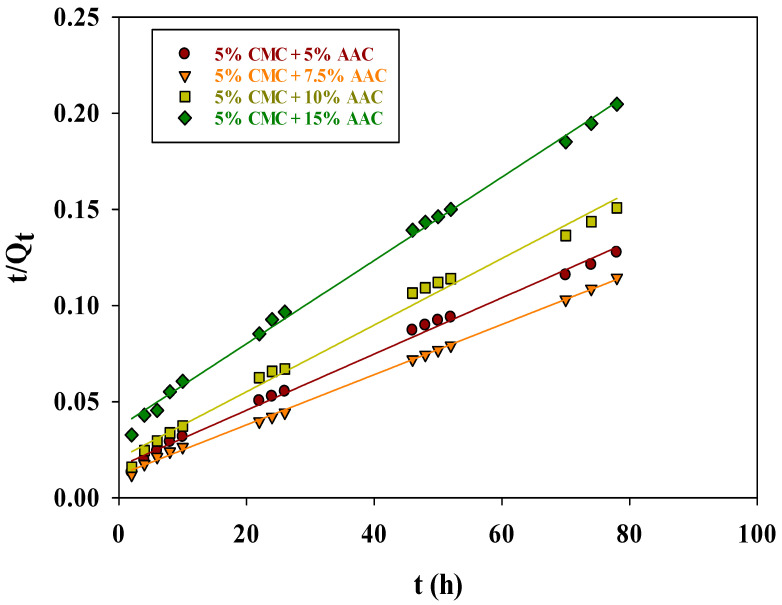
Pseudo-second-order kinetic model for adsorption of MB on CMC/AAc hydrogel.

The plot of (t/Q_t_) vs. (t) (Figure 9) provided the uptake and rate constant values, which can be derived from the slope and intercept of the line, respectively. A summary of these values, including k_2_ and Q_e, cal_ values, and correlation coefficients (R^2^), is presented in Table 2.

The k_2_ values indicate that the initial adsorption of MB was rapid due to the high MB concentration on the hydrogel surface. Over time, the rate of adsorption slowed as pore-filling diffusion occurred. The high R^2^ values (0.9950, 0.9988, 0.9904, and 0.9977 for 5%, 7.5%, and 15% AAc concentrations, respectively) confirm the model’s applicability. The calculated Q_e_, _cal_ values (666.67, 769.23, 588.24, and 454.55 mg/g) were higher than the experimental Q_e, exp_ values (611, 681, 517, and 381 mg/g) by 8.35%, 11.47%, 12.11%, and 16.18%, respectively. This reduced deviation, in comparison to the first-order model, implies that the pseudo-second-order model accurately represents MB adsorption kinetics on CMC/AAc hydrogels.

#### 2.9.3. Elovich Kinetics

Zeldowitsch’s Elovich kinetic model assumes that adsorbent surfaces are energetically heterogeneous and that there are no interactions between adsorbed species. The linearized form of this model is given by:

(3) $$Q_{t}=\frac{1}{\beta }\ln\left(\alpha \beta \right)+\frac{1}{\beta }\ln t$$

**Figure 10:**
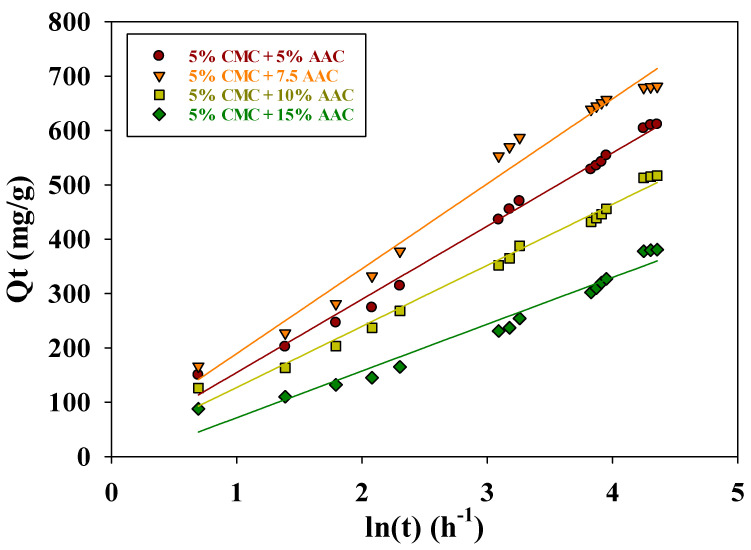
Elovich kinetic model for adsorption of MB on CMC/AAc hydrogel.

The Elovich coefficients α (mg g^−1^ h^−1^) and β (mg g^−1^ h^−1^) represent the initial adsorption rate and desorption coefficient, respectively. Additionally, the degree of surface coverage and activation energy for chemisorption are correlated with β. Table 2 lists the kinetic constants α and β, derived from the slope and intercept of the (Q_t_ vs. ln t) plot, respectively (see Figure 10).

The higher α values (156.35, 193.43, 128.30, and 101.69 mg g^−1^ h^−1^) compared to β values (0.0074, 0.0064, 0.0089, and 0.0116 mg g^−1^ h^−1^) indicate a higher adsorption rate than desorption, which demonstrates the viability of the process. When comparing Lagergren’s pseudo-first-order and pseudo-second-order models, the pseudo-second-order model better describes the adsorption of MB on CMC/AAc hydrogel from an aqueous solution.

### 2.10. Diffusion Models in Adsorption

The mechanisms of MB adsorption onto CMC/AAc hydrogel from aqueous solution were studied using intra-particle and liquid film diffusion models.

#### 2.10.1. Liquid Film Diffusion Model

The liquid film diffusion model [58] applies when the adsorption rate is controlled by the slow movement of adsorbate through the liquid film around the adsorbent particles. The mathematical representation of this model is:

(4) $$\ln\left(1\;-\;F\right)=\;-k_{\mathrm{fd}}t$$

**Figure 11:**
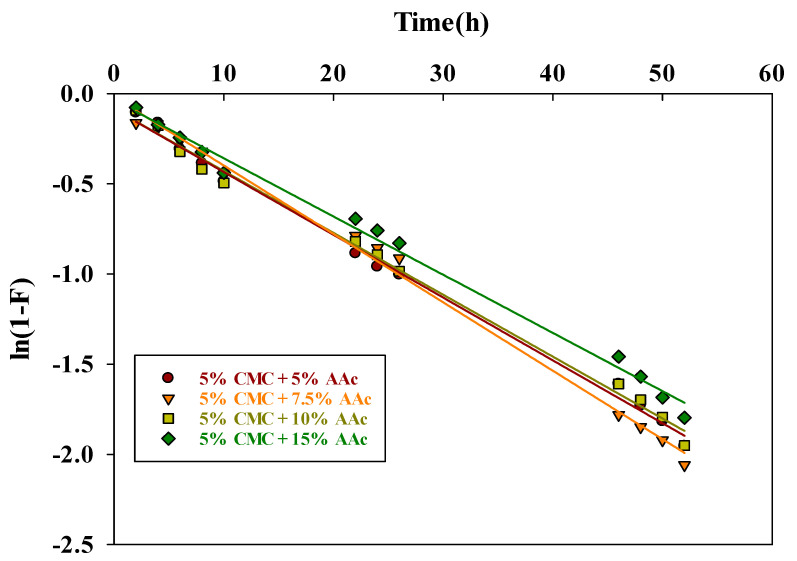
The liquid film diffusion kinetic plot for MB dye adsorption.

The film diffusion rate constant is represented by k_fd_, and the fractional attainment of equilibrium is given by F = Q_t_/Q_e_. A linear plot of ln(1−F) vs. t with a zero intercept indicates that adsorption kinetics are governed by diffusion through the liquid layer surrounding the solid adsorbents. Table 3 presents the values of the k_fd_ and R^2^, which were determined from the slopes of the straight-line plot shown in Figure 11.

The R^2^ values near unity suggested that the film diffusion model was well-fitting. However, the straight lines cross close to the origin, indicating that the only rate-limiting step may be resistance or film diffusion.

#### 2.10.2. Intraparticle Diffusion Model

In this model, the rate-determining phase may be the diffusion of adsorbate species into the pores of the adsorbent. The uptake solute by the adsorbent fluctuates in proportion to t^1/2^. The expression for the intraparticle diffusion model [59] is as follows:

(5) $$Q_{t}={\;k}_{\mathrm{ipd}}t^{\frac{1}{2}}+C$$

To calculate the intraparticle diffusion rate constant (k_ipd_) in mg.g^−1^ h^−1/2^ (see Table 3), we analyzed the slope of the plot of Q_t_ vs. t^1/2^, with (C) as the intercept. If the line passed through the origin, it suggested that intraparticle diffusion was the rate-limiting step; any deviation from the origin indicated the influence of film diffusion [60].

**Figure 12:**
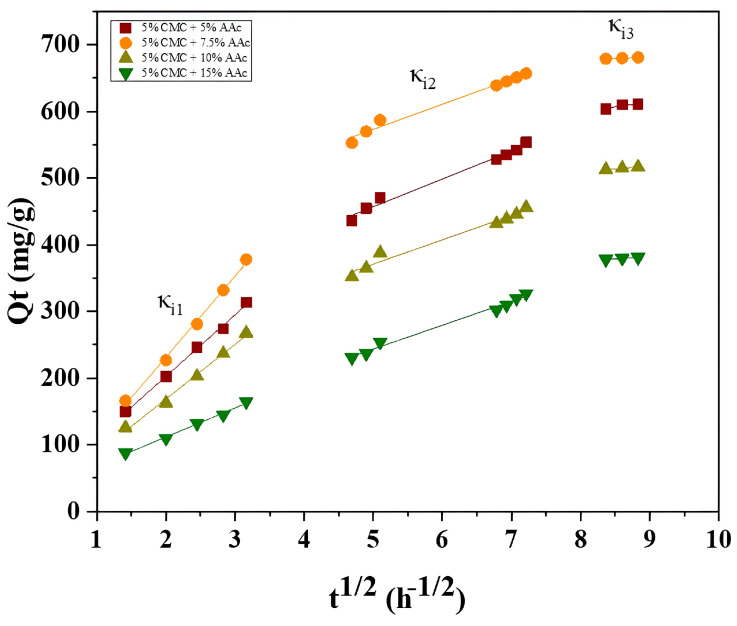
Intraparticle diffusion plots for the CMC/AAc hydrogels.

Figure 12 shows three linear segments in the plots, which represent three distinct diffusion stages of MB adsorption onto CMC/AAc hydrogel. The first stage involves rapid adsorbate transfer via boundary layer diffusion. The second stage, which is the rate-determining step, involves MB molecules diffusing into the hydrogel pores. The third stage pertains to the final adsorption at internal pore sites, ultimately reaching equilibrium. The adsorption rate constants follow the order κ_i1_> κ_i2_ > κ_i__3_, corresponding to the external surface, internal surface, and equilibrium stages. The pseudo-second-order kinetic model accurately describes the experimental results, indicating that both film diffusion and intraparticle diffusion play significant roles in governing the adsorption kinetics, which is consistent with previous studies [61].

### 2.11. UV-Visible Spectroscopy of MB Adsorption

**Figure 13:**
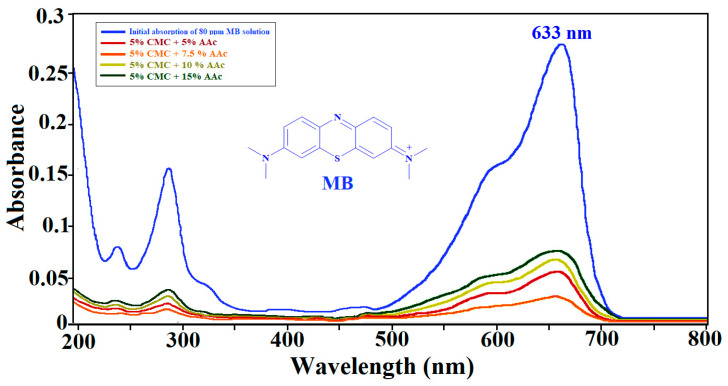
UV–visible spectra for MB adsorption by CMC/AAc hydrogels.

Using the Beer–Lambert law, the concentration of MB was determined at 663 nm [62]. After 78 h of equilibrium adsorption, Figure 13 confirms MB absorption by the hydrogel, presenting the UV–visible absorbance spectrum of the initial MB solution (80 mg/L) alongside the spectra of the aqueous phases surrounding the CMC/AAc hydrogels.

### 2.12. Mechanism of MB Adsorption

**Figure 14:**
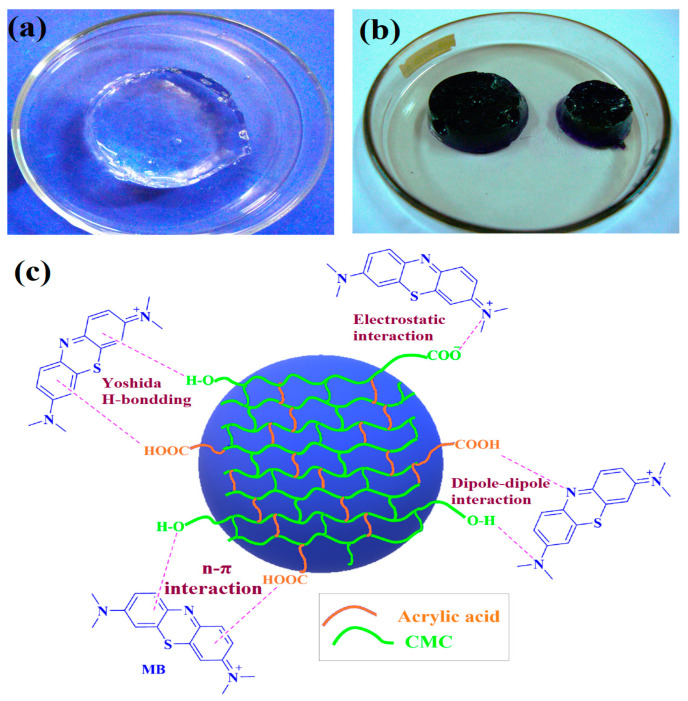
CMC/AAc hydrogel (**a**) before and (**b**) after MB dye adsorption and (**c**) possible adsorption mechanisms.

Figure 14 illustrates the adsorption mechanism of the CMC/AAc hydrogel adsorbent. Initially, the hydrogel swelled significantly, achieving an exceptionally high swelling ratio while maintaining its shape. This extensive swelling allowed the polymer network to fully expand, creating large channels for the diffusion of dye molecules.

When the hydrogel adsorbent was introduced into an MB solution, the MB molecules diffused into the hydrogel and rapidly bound with carboxylate and hydroxyl groups [63]. The high swelling also exposes sufficient active sites, resulting in high adsorption capacity [64]. Methylene blue (MB), a cationic organic dye, carries positive charges on its surface, while CMC/AAc hydrogels contain numerous electron pairs, leading to electrostatic attraction between MB and the hydrogel. Additionally, the aromatic rings in the chemical structures of both MB and the hydrogel facilitate π-π stacking interactions [65].

### 2.13. Comparative Study

Table 4 shows the comparative adsorption capacities of different adsorbents for removing MB from water. Recent studies have extensively investigated the adsorption capacity (Q_m_, mg/g) of methylene blue (MB) on various adsorbents.

Our current study on CMC/AAc gamma radiated hydrogels revealed a significantly higher adsorption capacity of 681 mg/g for MB dye. This superior capacity underscores the efficiency of CMC/AAc gamma-radiated hydrogels, marking them as a highly effective material for wastewater treatment applications and surpassing many previously reported adsorbents.

### 2.14. Desorption Mechanism of MB from CMC/AAc Hydrogels

Desorption of MB from the hydrogel was accomplished using 2M HCl, resulting in a desorption efficiency of 95%. The hydrogels were then regenerated through alkali treatment and reused for MB adsorption [72]. The effectiveness of the acidic solution in disrupting interactions between MB and the hydrogel matrix was evident, demonstrating the high efficiency of MB desorption from CMC/AAc hydrogels using 2M HCl [73]. Protonation of carboxyl and hydroxyl groups reduced electrostatic interactions, while excess H^+^ ions disrupted hydrogen bonds, facilitating the release of the dye [74]. The HCl solution also weakened van der Waals forces and increased hydrogel swelling, which enhanced dye diffusion [75]. Desorption kinetics followed a pseudo-second-order model, indicating variable desorption rates due to the heterogeneous nature of the hydrogel surface [76]. Higher HCl concentrations and longer contact times improved desorption efficiency, particularly in hydrogels with higher initial dye loading. The high desorption efficiency implies that CMC/AAc hydrogels are feasible and economical for wastewater treatment, allowing multiple adsorption–desorption cycles [77]. Understanding these mechanisms is crucial for optimizing hydrogel performance in environmental applications.

## 3. Conclusions

This study successfully synthesized CMC-based hydrogels grafted with AAc using gamma radiation polymerization. Various molar ratios of AAc to CMC (5:5, 5:7.5, 5:10, and 5:15) were treated with 37% NaOH and subjected to different radiation doses (1, 2, 3, 5, 10, and 15 kGy). A 5 kGy dose was identified as optimal for hydrogel preparation. Swelling studies indicated that the 5–7.5% hydrogel achieved the highest swelling ratio, while higher AAc content (10–15%) led to a decrease in swelling. FTIR spectroscopy confirmed the interaction between AAc and CMC, indicating successful hydrogel formation. Differential scanning calorimetry (DSC) showed that higher AAc content raised both the glass transition and decomposition temperatures. Grafting AAc disrupted the crystalline structure of CMC, enhancing the hydrogel’s thermal stability. Increased cross-linking reduced pore size and improved AAc grafting. The swelling kinetics followed Schott’s pseudo-second-order model. The impact of cross-linking on MB adsorption was also examined, revealing an increased adsorption capacity with 5–7.5% AAc content, while further cross-linking led to a more rigid structure that reduced dye penetration. The maximum adsorption capacity of 681 mg/g was achieved by the 5:7.5 hydrogel for a dye solution with an initial concentration of 80 mg/L. Kinetic analysis indicated non-uniform physisorption on heterogeneous surfaces, with adsorption governed by both intraparticle and film diffusion mechanisms.

Future research will focus on optimizing synthesis parameters to enhance adsorption capacity, while also analyzing desorption mechanisms, morphology, and structural stability using X-ray diffraction (XRD) and field emission scanning microscopy (FE-SEM). Exploring alternative monomers and cross-linking agents may yield more efficient and versatile hydrogel systems. Further evaluations will also be needed to assess the overall effectiveness of the synthesized hydrogels in environmental applications.

## 4. Materials and Methods

### 4.1. Materials

Carboxymethyl cellulose (purity 99.5%) was procured from Sigma-Aldrich, Bangalore, India, and used as received without further purification. The reagents used in this investigation were all of commercial quality, including acrylic acid (purity 99.5%, JDH, China), potassium hydroxide (purity 84%, Sigma-Aldrich, Bangalore, India), methanol (assay 99.8%), acetone (assay 99.5%, Merck, Germany), and methylene blue (purity ≥95%, Sigma-Aldrich, Bangalore, India).

### 4.2. Preparation of CMC/AAc Hydrogels

A 5% carboxymethyl cellulose (CMC) slurry was prepared in distilled water using a mechanical stirrer at room temperature. Acrylic acid, in varying concentrations (7.5% to 15.0%), was then incorporated into the slurry, followed by partial neutralization with potassium hydroxide (KOH). The mixture was transferred into sealed glass test tubes and exposed to γ-irradiation using a Co-60 source at doses of 1, 2, 3, 5, 8, and 10 kGy, with a dose rate of 3 kGy/h at approximately 27 °C. The irradiated CMC/AAc hydrogels were segmented into small pieces and dried in a vacuum oven until they reached a constant weight to assess their gel fraction and swelling properties.

### 4.3. Degree of Grafting Determination

The hydrogel samples were dried to a constant weight and extracted with methanol for 24 h to remove polyacrylic acid homopolymer. After extraction, the samples were washed thoroughly with distilled water and acetone to remove residual solvents, then dried and weighed. This process was repeated several times per sample for accuracy, and the average weight of the swollen samples was recorded. The degree of grafting was calculated using the following formula:

Degree of grafting (Wt%) = [W_1_/W_i_] × 100 Degree of grafting (%) = (W_i_W_1_) × 100 (6)

where (W_1_) is the weight of the dry sample after methanol extraction, and (W_i_) is the initial weight of the dry sample.

### 4.4. Gel Fraction Determination

The dried CMC/AAc hydrogel samples were soaked in distilled water for 24 h to remove soluble components. The extracted hydrogels were then dried in a vacuum oven to a constant weight. This process was repeated several times, and the average weight was recorded. The gel fraction was calculated using the following formula:

Gel fraction (%) = [W_1_/W_i_] × 100 Degree of grafting (%) = (W_i_W_1_) × 100 (7)

where (W_1_) and (W_i_) denote the water extracted dried and the initial weight of the samples, respectively.

### 4.5. Measurement of Water Absorbency and Equivalent Water Content

The water absorbency and equivalent water content (EWC) of CMC/AAc hydrogels were measured using the gravimetric method. Dried samples were soaked in distilled water at room temperature (~27 °C) until fully swollen. The swollen samples were then blotted with soft tissue paper to remove surface water and weighed. This process was repeated several times and average weight was recorded. The water absorbency and EWC were calculated using the following formula:

Water absorbency (g/g) = [W_2_ − W_1_]/W_1_ (8)

And EWC (%) = [W_2_ − W_1_]/W_1_ × 100 Degree of grafting (%) = (W_i_W_1_) × 100 Degree of grafting (%) = (W_i_W_1_) × 100 (9)

where (W_1_) and (W_i_) represent the weights of the swollen and dried samples, respectively.

### 4.6. Characterization

Fourier-transform infrared (FTIR) spectra of CMC/AAc hydrogels were obtained using a Shimadzu IP Prestige-21 FTIR Spectrometer(Shimadzu corporation, Kyoto, Japan).

Differential scanning calorimetry (DSC) was performed using T Zero thermally sealed alumina pans on a calcium oxalate calibrated TA Instruments SDT Q-600 (New Castle, Delaware, USA). Samples were heated from 25 °C to 550 °C at a rate of 10 °C/min in a nitrogen atmosphere (100 mL/min) to prevent oxidation or degradation.

### 4.7. Biodegradability

The biodegradability of a CMC/AAc hydrogel film was evaluated in a composting environment maintained at 27–30 °C, with a pH range of 6.5–8.0 and approximately 60% moisture content. These conditions are highly conducive to the growth of microorganisms responsible for biodegradation.

### 4.8. Dye Adsorption Assessment

The CMC/AAc hydrogels, synthesized using 5 kGy gamma radiation, were immersed in 250 mL MB solution (initial concentration: 80 mg/L). Changes in dye concentration were monitored with a UV–visible spectrometer (UV-1800 Series, Shimadzu) (Shimadzu corporation, Kyoto, Japan). The adsorption capacity at time (t), denoted as Qt (mg/g), was calculated using the following equation:

(10) $$Q_{t}=\frac{(C_{o}-\;C_{t})V}{W}\mathrm{Degree}\;\mathrm{of}\;\mathrm{grafting}\;(\%)=(W_{i}W_{1})\times 100\mathrm{Degree}\;\mathrm{of}\;\mathrm{grafting}\;(\%)=\;(W_{i}W_{1})\times 100$$

where C_o_ and C_t_ are the initial and time (t) dye concentrations (mg/L), respectively, (V) is the solution volume (L), and (W) is the dry weight of the hydrogel (g).

## Figures and Tables

**Table 1:** Glass transition (T_g_), crystallization (T_c_), melting (T_m_), oxidation (T_oxi._), and decomposition (T_d_) temperatures of CMC, AAc, and cross-linked CMC/AAc hydrogels calculated from DSC data.

Samples	T_1_^a^	T_g_	T_c_	T_m_	T_oxi_	T_d_
CMC	231.49	228.73	293.32	322.64	n. d	403.58
AAc	242.01	242.01	272.05	287.84	n. d	451.38
5% CMC + 5% AAc	237.84	285.34	356.16	389.2	436.71	467.9
5% CMC + 7.5% AAc	238.76	290.41	357.99	396.01	438.7	468.06
5% CMC + 10% AAc	241.76	291.58	359.65	412.55	439.53	469.88
5% CMC + 15% AAc	243.67	292.82	360.06	425.15	446.99	473.62

T_1_ ^a^: Refers to bound water removal temperature; T_g_: Glass Transition temperature; T_c_: Crystallization temperature; T_m_: Melting temperature; T_oxi_: Oxidation temperature; T_d_: Decomposition temperature.

**Table 2:** Adsorption kinetic parameters for the adsorption of MB on hydrogels.

Hydrogel Composition	Pseudo-First-Order Kinetic Model				Pseudo-Second-Order Kinetic Model			Elovich Kinetic Model		
	Q_e_, _exp._ (mg/g)	Q_e_, _cal_ (mg/g)	k_1_ (h^−1^)	R^2^	Q_e_, _cal_ (mg/g)	k_1_ × 10^−4^ (h^−1^)	R^2^	α (mg. g^−1^ h^−1^)	β (mg. g^−1^)	R^2^
5% CMC + 5% AAc	611	448.85	0.0387	0.9865	666.67	1.38	0.9950	156.35	0.0074	0.9911
5% CMC + 7.5% AAc	681	533.84	0.0585	0.9895	769.23	1.42	0.9988	193.43	0.0064	0.9783
5% CMC + 10% AAc	517	373.34	0.0341	0.9831	588.24	1.4	0.9854	128.30	0.0089	0.9879
5% CMC + 15% AAc	381	303.46	0.0316	0.9934	454.55	1.31	0.9977	101.69	0.0116	0.9612

**Table 3:** Adsorption diffusion parameters for the adsorption of MB on hydrogels.

Hydrogel Composition	Liquid Film Diffusion Model			Intraparticle Diffusion (mg.g^−1^ h^−1/2^)					
	K_fd_	Intercept	R^2^	R^2^	R^2^	R^2^	κ_i1_	κ_i2_	κ_i3_
5% CMC + 5% AAc	−0.08598	0.99553	0.9950	0.9970	0.9860	0.8601	92.2542	41.6503	15.0960
5% CMC + 7.5% AAc	−0.01859	0.99507	0.9988	0.9962	0.9841	0.9999	121.473	37.6668	4.2993
5% CMC + 10% AAc	−0.08519	0.99464	0.9854	0.9935	0.9731	0.9999	82.0731	36.4603	8.5986
5% CMC + 15% AAc	−0.03512	0.99362	0.9977	0.9940	0.9883	0.9671	43.4481	35.7869	6.4586

**Table 4:** The MB adsorption capacity of hydrogels compared with other adsorbents.

Adsorbent	Methods	Morphology	Adsorption Capacity (mg/g)	Reference
Alkali-modified corn straw biochar	Pyrolysis	Rough surface and porous structure	290.71	[66]
CMC-g-Poly (MAA-co-AAm)/cloisite 30B	Free radicle polymerization	Porous structure	77.51	[54]
Starch/poly (acrylic acid)-based hydrogels	Free radicle polymerization	Amorphous porous structure	66.7	[67]
MIL-100(Fe)/cellulose	Nanocomposite	Porous hexagonal structure	384.62	[68]
Lignin nanoparticles	Hydrotropic	Microporous structure	127.91	[69]
Acrylic acid-chitosan hydrogel	γ- radiation polymerization	Porous structure	322	[70]
Acrylic acid-grafted-starch	γ- radiation polymerization	Porous structure	576	[71]
Carboxymethylcellulose-Acrylic acid	γ- radiation polymerization	Porous structure	681	This study

## Data Availability

The data presented in this study are openly available in the article.
